# Editorial

**Published:** 1916-01

**Authors:** 


### EDITORIAL

The Atlanta Journal-Record of Medicine has its Business Office at 23 West Alabama Street where all business communications should be sent.

Remittances are payable to The Journal-Record of Medicine.

Concerning Original Communications and Editorial matters, address Richard R. Daly, M. D., 708 Empire Life Building, Atlanta, Ga.

Reprints of Original Articles will be furnished at cost price. Requests for them had best be made on the Manuscript.

Upon request, we will present, postpaid, to each contributor of an original article, twenty copies of The Journal-Record of Medicine containing such article.

---

### WHO LEADS SHOULD KNOW THE WAY.

The successful leaders of men in the world have been sought and followed because of their known merit. On the other hand, the self-constituted heads of the processions may have worn the shakos and heard the applause from the sidewalks, but the parades have often broken up at the cemeteries.

In the Journal of the A. M. A. for January 1, 1916, there appeared the New Year's greeting to the Editor of this Journal. It is accepted in a kindlier spirit than it was sent. All good things are welcome and even some things in themselves distasteful, from which benefit may be derived, are received with thanks. It is not quite clear whether this was intended as a purge or a tonic and so we shall not say what the result has been. A simple acknowledgment should be enough and would suffice were it not that publicity has been given to the event without our acquiescence, and comment seems necessary.

The editorial in the Journal of A. M. A. was headed "Whose Bread I Eat, His Song I Sing." It considered an editorial in the November number of this Journal entitled "In Fairness." It spoke of our having published the United States Department of Agriculture press bulletin in which was a list of "other preparations against which the government's charge that they were falsely or fraudulently labelled was sustained by the Federal courts." It went on to say that in the next issue, we had "apologized" for including a certain preparation in this list and to prove it, quoted us as follows:

> "In our September issue, Gray's Glycerine Tonic Comp. was inadvertently included in a list that seemed to be under the ban of the Government and very likely an injustice has been done the Purdue Frederick Co. which we desire to undo as far as possible."

It proceeded to ask if this preparation "were fraudulently exploited, and the government and courts have so declared it, why is it necessary for the editor of a medical journal to apologize to his subscribers for having told them so?" It answers its own riddle and says truly enough "That the only reason that occurs to us is expressed in the caption of this article." The caption is the kindly intimation that the Journal-Record can sing for its bread which is better than snarling or whining for it of course, and by the way, we shall be glad to share a happy crust with a brother who is hungry and depressed.

The Journal-Record is not responsible for the limitation of reasoning faculty the gentleman announces. If the Editor of The Journal of The A. M. A. had asked us the question by mail, we should have told him that the reason we spoke "In Fairness" which headed our editorial was that we have a letter from the firm which is now getting gratuitous advertising, showing us that their representative had agreed with the government that a certain feature of the label would be changed although he did not admit even then it was false and fraudulent; showing that the label had been changed in accordance with the agreement, and showing that the self-same product was now on the market with government's approval or at least without government's objection after investigation. These transactions had been consummated before we published the list. The firm called our attention to the matter and we played fair so far as we were able. It was time to apologize to our readers for our error in the information our columns contained. This is a small point that the A. M. A. gentleman has overlooked. We do need our subscribers and we can hold them only by telling them the truth as we see it. We could get along for an issue or two without any one advertiser or all of them for that matter, though we would not be understood as wanting to defy or annoy anyone who has legitimate use for our space.

Another point is this: that we attempt only to place our matter before our clientele and we then rely upon the judgment of each reader to do what he thinks best about it. We believe that each man is competent to practice medicine and to select his standards of morality and his drugs without our dictation. We said just that in the same editorial from which the above quotation was taken and we added:

> "To the basic principles of medical ethics we are hearty subscribers. They may be summed up with the terms common decency and fair play, or if there are any outside of these, we are not familiar with them."

This did not attract the attention of our critic; it could not have done so, for we are sure he would not allow a partial and apparently damaging quotation to appear inadvertently in his paper for the reasons that such things are not looked upon as nice and that they call for apologies which are adhorrent to some minds.

Thus we can not even intimate that the writer of the catchy phrase caption does know of principles of ethics outside our limit. We do not wish to hint that he should search for them.

There is a gracefulness of style in the complaining commentary that is marred by an acerbity of thought which suggests a sort of temperamental acidosis. There are implied threats and actual sneers and they do not look well coming from a leader of any sort, besides which they are not good writing such as might characterize a leading journal. A little element of the better-than-thou idea appears and mixed with this Phariseeism is a symptom of megalomania that would make the teacher determine more things for us than we all like. At the same time, both the precept and the example are vague, for is it not true that The A. M. A. is in the Courts? Our local newspaper said it was and that its organic construction and subsequent administration have clashed because of the management of its affairs. This is not an editorial function and the writer who filled the half column of space we have before us may have had nothing to do with it. If so he has been inadvertent and has regarded his Ter Sanctus carelessly. It would look better before the people were it immaculate and unsullied.

Perhaps an injection of the vitamine of good-fellowship would help and then our Brother of the Cloudy Brow who has hidden his good nature behind an austere demeanor might see that we all want to do right, that we all are honest and sincere, that we have different views of the same things which when brought together will give contour and depth and an approximation to the truth that one alone can not get, that to be dictatorial to others is not good form in teaching and that in our work together or apart there might evolve the perpetuation of each others' friendship and each others' love.

The Journal of The Medical Association of Georgia has copied the criticism of the A. M. A. verbatim as an editorial in January, giving proper credit.

We congratulate it upon having an editorial.

Since the foregoing was written, the exchanges have brought two editorial opinions which are quoted here, and a contribution from a subscriber which is published in another section. These show that in the minds of at least three men there has been vexation of spirit induced by the mentnal invagination of the distinguished A. M. A. victim of coacoethes carpendi.

---

### "UNDER WHICH KING, BEZONIAN?"—'TOTHER OR WHICH?

(Southern Practitioner, January, 1916.)

On December 24th, we received a communication bearing the letterhead of the American Medical Association, dated December 21st, 1915, and signed American Medical Association, Will C. Braun, the first paragraph being as follows:

"Dear Doctor Roberts:

"You are to be congratulated on the splendid spirit of co-operation you are showing by maintaining membership in your

County and State Medical organizations. This demonstrates your interest in the betterment of conditions pertaining to your profession and its opportunities."

In the same envelope was enclosed several "leaflets" from one of which we make this extract, it being taken from The Journal of the American Medical Association of November 13th, 1915:

"In the Propaganda Department this week (see p. 3), we print an editorial on advertising copied from the New York State Journal of Medicine. The conclusion is couched in strong language—but not a whit too strong:

" 'When an exploiter places on the market a nostrum of no intrinsic value (excepting the bottle and cork) for which he claims virtues which it does not possess . . . he robs the purchaser of his money and health, thereby becoming a thief of the most despicable order. Any medical journal printing the fraudulent claims contained in the advertisements of the nostrums condemned by the Council on Pharmacy and Chemistry is an accessory to this act of thievery and the subscriber to such journals voluntarily assumes the position of an accomplice."

This leaflet, or more properly a "pronunciamento" from the "Propaganda, (we came very near making that last syllable der), goes on further to arraign our esteemed and valued contemporaries The N. Y. Medical Record, the N. Y. Medical Journal, and the Therapeutic Gazette for admitting certain advertisements to their pages. We cannot but infer that this "progageezer" would place us in a similar class, as we have been pleased for years past, to place before our readers, the advertising matter of a number of "proprietaries" now banned by the Propaganda, but by many of our readers and ourselves regarded as not only valuable, but well-nigh "standard."

We do not like to be called a thief, or classed as one, for we do not think we have ever stolen anything—at any rate, it has not been proved on us; nor did we secure our degree of M.D., as a Regular Doctor of Medicine by fraud and falsehood!

Furthermore, many of the advertisements appearing in our pages that so offend the "water-cure wizard of Dearborn Street and his satellites," regularly appeared in the pages of the Jour.

of the A. M. A., when it was under the guidance of such grand and noble colleagues as N. S. Davis, the "Father of the Association," Jno. C. Culbertson, Jno. H. Hollister, and Jno. B. Hamilton. Oh yes, any dog may bark at me, but then I can feel a little doubtful and chary when he licks my hand.

Oh, well! There is a better day coming—and everything comes to him who will wait. A recent legal ruling as to the standing of the A. M. A., under its present mismanagement may have some effect, and although we are not a lawyer, we are satisfied in the belief that a libel suit would stand—in re The N. Y. State Journal of Medicine and the "J. M. A." and the above quotation therefrom. The small perSimmons and his clique are on thin ice, if they only knew it.

---

### IS THE A. M. A. AUTOCRATIC?

(The Medical Herald, January, 1916.)

Word comes of the necessity of reorganization of the A. M. A. The Supreme Court of Illinois has so declared. It has some aspects of a brush. That the methods and rulings and tendencies of the association has not met the general approval of the medical profession goes without saying. It cannot be denied. The necessity of organization is recognized, the desire for it is universal, but—cannot it not be more democratic in tenor? The prominent tone primarily of the association was one of education, elevating the profession, educating the people to a proper appreciation of the medical profession. In the opinion of many the attitude, in recent years, of the association has been somewhat Czarish. The decisions of the council have not met general approval; the decrees of certain ruling spirits have been just a little oppressive concerning school matters, journal relationship, opinions of individuals or of medical societies not affiliating with the A. M. A. There is a little tendency to force matters to meet the dictates or opinions of some officers of the association, to such a degree as to dwarf the individuality of members or societies or to jeopardize the very existence in independent journals. This tendency toward the trust aspect of the association cannot continue without impairing the good fellowship and esprit de corps of the profession, nor is it approved by the profession at large. It is to be hoped, in the reorganization, much of this unfortunate leaning toward the autocratic may be eliminated, and the hand of good fellowship and unity of purpose between the rank and file of the ruled and the ruler be maintained. May the gulf between the directors room and the members be entirely eliminated.—J. M. B.

---

### RAILROAD FIRST-AID.

In these times when ever so many things are being foreseen and having preparations made for them, the subject of first aid to the passengers on railway trains seems to have been omitted. At all events it is not a common thing to find in a passenger coach or Pullman the things necessary to assist a passenger who may be suffering with faintness, nausea or cramp; and when accidents occur to the trains, the people have to wait until the nearest doctors can be brought to them.

Axes and hammers with which to make escapes, emergency levers with which to stop the trains, inclosed vestibules with which to prevent lurches and fatal falls have long been provided, but the sinple things a surgeon or one who knows something of first aid would need are not at hand.

Many of us have been in or near train accidents and found ourselves helpless because our kit bags were not with us. We have had to stand and wait. While it may be true in some instances that "He also serves who only stands and waits," it is an aggravating sense of uselessness and futility that a physician feels when he is prevented from doing things for lack of first aid material.

Of course, there are always some things a skilled man can do to help if he has nothing more than his fingers to work with, and he will be found doing them, too; but what a waste of time there is when he has to confine his attention to one patient while another may be bleeding to death. A proper kit would save lives.

It is true that a surgeon is not always at hand, but in these days when sanitation and first aid are being taught in the schools we can expect some intelligent person to be present and able to use sinple and well-arranged remedies.

It would not cost so very much to equip every car with one first aid package in each end of the aisle. The pack could be arranged by the road's surgeon and directions of simple and plain character could be printed so as to make the help available.

In addition each conductor might be furnished with a small supply of emergency drugs to meet the conditions of prostration such as often arise in travelling and these might be dispensed by some one who knew how without putting further responsibility upon the conductor.

The railroad that begins something of the kind will earn enough good will from its patrons to repay many times over for the outlay and we are sure that it's example would be followed by other roads.

It is becoming the policy of the people to get better acquainted with the roads and in a better manner. The prosecutions are giving place to common sense agreements and the old idea that anything to beat the roads is good has been dying out. Men are coming to understand that passes and the system of riding for nothing is not a good paying proposition for the roads or for the public. On the other hand, the policy of many roads seems to be to tell the people more of what is going on and what they have a right to know about. When an accident occurs to make trains late, the conductors have been told to act like sensible humans and relieve the irritated curiosity delayed passengers naturally feel. A mutual courtesy is developing and it is bound to be of value to both elements.

The introduction of suitable first aid arrangements in all the passenger coaches would go a long way to further the good feeling as well as to save the lives upon which both the public and the roads depend.

We have endeavored to get into touch with our subscribers and to please them so far as we were able with the character of our articles. It is the policy of the Journal-Record to place the best it has before its clientele and to deal openly with them in every way.

In order that this plan can be furthered and perfected, we are inviting correspondence from our subscribers upon any subject that might interest them. We shall publish such letters as the writers desire and give names or not as the authors indicate. While we are always looking for commendation, we know that we can not always deserve it and we do not expect that the letters shall invariably praise. Indeed, constructive criticism is the best kind.

In addition to this, it often happens that a man has an idea of a remedy that would do others good, but he doesn't care to make a formal article of its description. A letter to the Editor would convey all he had to say and do it better than anything else. It would be easy, too. We should like to have that intimate touch with all our subscribers and readers. We should like to have the Journal belong to its readers in just that sense and we believe it would do everyone good.

This invitation is sincere and cordial and it means that every letter will be treated with respect. If anyone wants to disapprove and does so genially and constructively, we shall publish the letter just as freely as if it were praise, no matter if it should hit us pretty hard.

Of course anonymous letters will not be considered.

---

### FULTON COUNTY MEDICAL SOCIETY.

At the Annual Meeting of the Fulton County Medical Society, the retiring President, Stewart R. Roberts, gave an inspiring address containing the expression of high ideals as to the relations of physicians to themselves and to their work.

The election of officers for 1916 followed and these gentlemen were elected: President, Dr. W. A. Selman; Vice-President, Dr. R. B. Ridley, Jr.; Secretary-Treasurer, Dr. Walpole Brewer; Member of Board of Censors, Dr. H. R. Donaldson.

